# Clinical and radiomics integrated nomogram for preoperative prediction of tumor-infiltrating lymphocytes in patients with triple-negative breast cancer

**DOI:** 10.3389/fonc.2024.1370466

**Published:** 2024-03-19

**Authors:** Ling Hu, Peile Jin, Wen Xu, Chao Wang, Pintong Huang

**Affiliations:** ^1^ Department of Ultrasound in Medicine, The Second Affiliated Hospital, Zhejiang University School of Medicine, Hangzhou, Zhejiang, China; ^2^ Department of Ultrasound in Medicine, Hangzhou Women’s Hospital, Hangzhou, Zhejiang, China; ^3^ Research Center of Ultrasound in Medicine and Biomedical Engineering, The Second Affiliated Hospital of Zhejiang University School of Medicine, Zhejiang University, Hangzhou, Zhejiang, China; ^4^ Research Center for Life Science and Human Health, Binjiang Institute of Zhejiang University, Hangzhou, Zhejiang, China; ^5^ Department of Radiology, the Second Affiliated Hospital, Zhejiang University School of Medicine, Hangzhou, Zhejiang, China

**Keywords:** radiomics, tumor-infiltrating lymphocytes (TILs), breast cancer, triple-negative breast cancer (TNBC), ultrasound

## Abstract

**Objectives:**

The present study aimed to develop a radiomics nomogram based on conventional ultrasound (CUS) to preoperatively distinguish high tumor-infiltrating lymphocytes (TILs) and low TILs in triple-negative breast cancer (TNBC) patients.

**Methods:**

In the present study, 145 TNBC patients were retrospectively included. Pathological evaluation of TILs in the hematoxylin and eosin sections was set as the gold standard. The patients were randomly allocated into training dataset and validation dataset with a ratio of 7:3. Clinical features (age and CUS features) and radiomics features were collected. Then, the Rad-score model was constructed after the radiomics feature selection. The clinical features model and clinical features plus Rad-score (Clin+RS) model were built using logistic regression analysis. Furthermore, the performance of the models was evaluated by analyzing the receiver operating characteristic (ROC) curve, calibration curve, and decision curve analysis (DCA).

**Results:**

Univariate analysis and LASSO regression were employed to identify a subset of 25 radiomics features from a pool of 837 radiomics features, followed by the calculation of Rad-score. The Clin+RS integrated model, which combined posterior echo and Rad-score, demonstrated better predictive performance compared to both the Rad-score model and clinical model, achieving AUC values of 0.848 in the training dataset and 0.847 in the validation dataset.

**Conclusion:**

The Clin+RS integrated model, incorporating posterior echo and Rad-score, demonstrated an acceptable preoperative evaluation of the TIL level. The Clin+RS integrated nomogram holds tremendous potential for preoperative individualized prediction of the TIL level in TNBC.

## Introduction

Triple-negative breast cancer (TNBC), a distinct form of breast cancer, is identified by the lack of estrogen receptor (ER), human epidermal growth factor receptor type 2 (HER2), and progesterone receptor (PR) expression (1). TNBC has a higher rate of recurrence and metastasis than non-TNBC (1, 2).

Tumor-infiltrating lymphocytes (TILs) have proven to be a dependable predictor of treatment response and prognosis in cases of breast cancer (3, 4). TILs are primarily observed in cases of TNBCs (5, 6). In several previous studies, the presence of higher TILs in TNBC tumors was found to be associated with improved outcomes (5–9). The patients with higher TILs in breast cancer were shown to have a higher responsiveness to chemotherapy compared to the patients with lower TILs in breast cancer (10–12). In the past few years, there has been a growing emphasis on the utilization of immunotherapy, particularly the application of immune checkpoint inhibitors, owing to their favorable clinical outcomes observed in both early and advanced TNBC (13–15). It is anticipated that TILs will function as a prognostic indicator for the immunotherapeutic efficacy in patients receiving immune checkpoint inhibitors. At present, TILs are often assessed in histopathological slides obtained through invasive core needle biopsy, especially for neoadjuvant chemotherapy patients (3). However, the core needle biopsy has several well-known pitfalls, including the limited tissue sampling and heterogeneity in lymphocyte distribution, which may affect the accuracy of evaluating TIL level. Hence, it is imperative to develop a non-invasive imaging method allowing for visualization of the entire tumor to serve as an important supplement to evaluate TIL level.

Breast conventional ultrasound (CUS) is a real-time, non-radiative, cost-effective and commonly used diagnostic imaging method for breast tumors in clinical settings. Radiomics, a potentially valuable quantitative technique, involves the extraction of high-throughput imaging characteristics from medical images (16, 17), which has extensive application in classifying phenotypic subtypes and making prognostic predictions for solid tumors (18, 19). CUS-based radiomics has shown the potential to differentiate malignant and benign lesions (20) and evaluate the prognosis for patients with breast cancer (21, 22). However, limited research has been conducted on the efficacy of the CUS-based radiomics approach in differentiating high TILs from low TILs in TNBC. This research aims to investigate the potential of CUS-radiomics models for preoperative assessment of TIL level in patients with TNBC.

## Methods

### Patients

**Figure 1:**
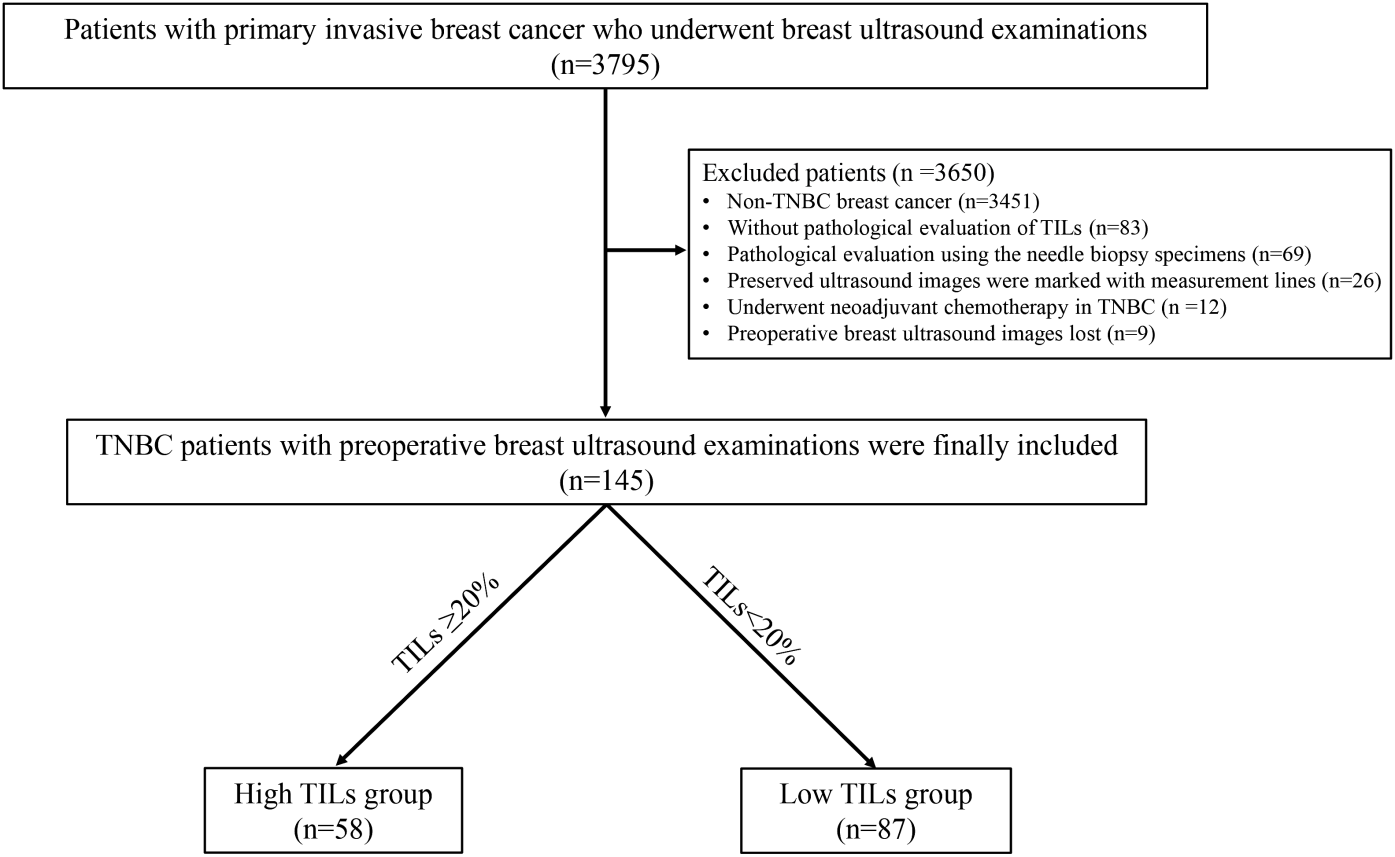
Flowchart of the patient inclusion.

**Figure 2:**
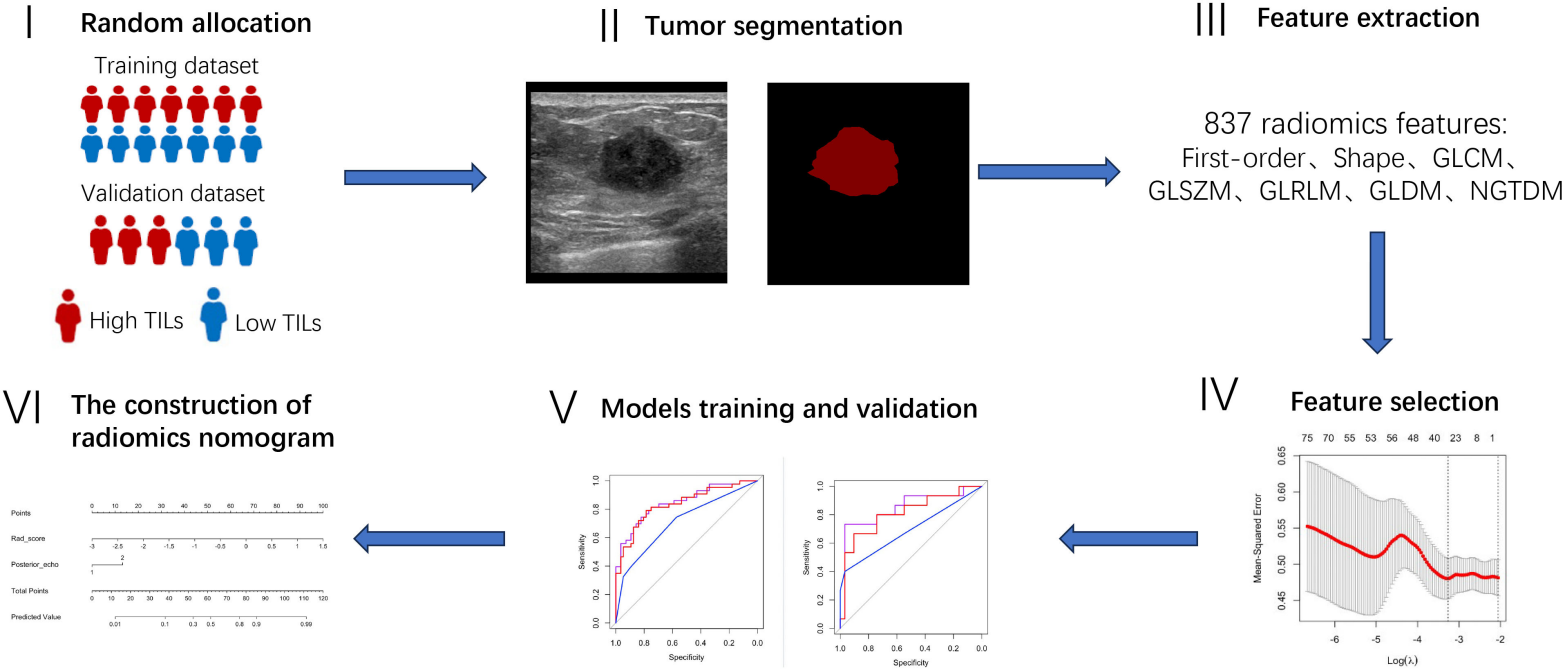
The flowchart of the study.

In the present study, we retrospectively enrolled primary invasive breast cancer patients in our hospital from 1 January 2017 to 30 October 2023 with baseline breast ultrasounds available. A total of 3795 consecutive patients were initially included who underwent breast ultrasound examinations and were confirmed with pathological diagnosis of primary invasive breast cancer. The criteria for exclusion were as stated below: (1) non-TNBC breast cancer proven by immunohistochemistry (IHC) staining (n=3451), (2) without pathological evaluation of TILs (n=83), (3) pathological evaluation using the needle biopsy specimen but not surgical resection specimens (n=69), (4) preserved ultrasound images were marked with measurement lines (n=26), (5) underwent neoadjuvant chemotherapy in TNBC before surgical resection (n =12), (6) preoperative breast ultrasound images lost (n=9). After these exclusions, 145 patients with TNBC were finally included. The detailed patient inclusion is illustrated in **Figure 1**. The framework of the current study is presented in **Figure 2**.

### Pathologic evaluation

The assessment of stromal TILs was conducted based on pathological hematoxylin and eosin (H&E) slides by pathologists according to published guidelines (23). The percentage of stroma in each breast cancer tumor was documented relative to the lymphoid cells (24). In this study, TILs refer to stromal TILs unless otherwise specified. Previous studies found that a percentage of TILs≥20% was associated with pathologic complete response in TNBC (25, 26). Thus, this study defined the percentage of TILs≥20% as a high TIL level.

### Ultrasound examination

Experienced board-certified radiologists performed all breast ultrasound examinations. Several ultrasound machines were used for conducting breast ultrasound examinations, including ESAOTE (MyLab 90 X-vision, Italy) and Logic E9 (GE Healthcare, USA) with high-frequency probes. Relevant images of the primary tumor were recorded during the examination, including both transverse and longitudinal views.

### Ultrasound characteristics evaluation by radiologists

Two experienced radiologists with ≥ 8 years of expertise in breast ultrasound retrospectively assessed the CUS characteristics of the index tumor for each patient. They were blinded to the ultrasound and pathologic reports. In the event of a discrepancy between the two observers, a consensus was reached following deliberation. The CUS characteristics were assessed for the following CUS features: shape, margin, orientation, echo pattern, posterior echo features, vascularity, and calcification.

### Tumor segmentation and radiomics feature extraction

To begin with, the patients were randomly assigned to either the training or validation group in a 7:3 ratio. Then, the jpg file format was used to save the ultrasound images, and subsequently, they were imported into the PyCharm software (2022.2 edition). Subsequently, a radiologist with ≥ 8 years’ experience in breast ultrasound manually delineated the region of interest (ROI), which was then verified by another radiologist with ≥ 8 years’ experience in breast ultrasound utilizing the Labelme software package (3.16.7). Neither of the radiologists had knowledge of the pathological findings while annotating the images, and any discrepancies were resolved through consensus reached via discussion. Radiomics characteristics were obtained from tumors following image processing using various filters through the utilization of the Pyradiomics package (Version 3.0.1) which were subsequently categorized into distinct classes, including 1) first-order features; 2) shape-based features; 3) high-order features, including GLCM, GLDM, GLRLM, GLSZM, and NGTDM. Finally, 837 radiomics features were extracted for this study.

### The establishment of clinical, Rad-score, and clinical features plus Rad-score integrated models

To begin with, we employed an independent t-test or Mann-Whitney U test to identify significant features exhibiting statistically meaningful differences (P<0.05). Afterwards, the least absolute shrinkage and selection operator (LASSO) was utilized for selecting coefficients that are not zero through cross validation of 10-fold. Lastly, the Rad-score was computed using the chosen features. The detailed formulation of the Rad-score can be found in the **Supplementary Materials**.

The baseline clinical features and Rad-score were compared using univariate analyses between the low TILs and high TILs groups (**Table 1**). Then, multivariable logistic regression models were constructed by including variables that had a significance level of p <0.05 in the univariate analyses in both the clinical features model and clinical features plus Rad-score (Clin+RS) integrated model. The model’s ability to discriminate was assessed through analysis of the receiver operating characteristic (ROC) curve, while the accuracy of the models’ predictions was measured via calculating the area under the curve (AUC) in the training dataset and validation dataset.

**Table 1:** Clinical characteristics and Rad-score in 145 patients with TNBC, stratified by TIL level.

	Low TILs (n=87)	High TILs (n=58)	P value
Age (y), mean ± SD	55.8 ± 10.3	53.0 ± 10.2	0.112
Size (mm), median	20.0 (14.0, 25.0)	20.5 (16.0, 25.0)	0.148
Shape			0.004
Oval/round	34(39%)	37(64%)	
Irregular	53(61%)	21(36%)	
Orientation			0.026
Parallel	63(72%)	51(87.9%)	
Not parallel	24(28%)	7(12.1%)	
Margin			<0.001
Well-defined	16(18%)	27(46.6%)	
Ill-defined	71(82%)	31(53.4%)	
Echo pattern			0.030
Complex cystic-solid	2(2%)	7(12%)	
Hypoechoic	85(98%)	51(88%)	
Posterior echo			<0.001
Enhancement	7(8%)	23(40%)	
No/Shadowing	80(92%)	35(60%)	
Calcification			0.400
Absent	57(65%)	34(59%)	
Present	30(35%)	24(41%)	
Vascularity			0.415
Absent	26(30%)	12(21%)	
Internal vascularity	37(42%)	30(52%)	
Vessels in rim	24(28%)	16(27%)	
Rad-score, median	-0.79(-1.28, -0.45)	-0.12(-0.44, 0.47)	<0.001

### Statistical analysis

Statistical analyses were conducted utilizing SPSS software (version 26) and R software (version 4.2.1). Quantitative variables were expressed as mean ± SD or medians (25th percentile, 75th percentile). The normality of the data was evaluated using the Kolmogorov–Smirnov test. To analyze quantitative variables, either an independent samples t-test or a Mann–Whitney U-test was conducted, while categorical variables were analyzed using either a chi-square test or Fisher’s exact test. The models were assessed for their diagnostic accuracy using ROC analysis. To compare the ROC curves, DeLong’s test was employed. Moreover, calibration curves were performed to evaluate the predictive performance. Furthermore, decision curve analysis (DCA) was conducted to evaluate the clinical usefulness. A statistically significant result was defined as P < 0.05 (two-sided).

## Results

### Clinical data

In this study, 145 female patients with TNBC were finally included, including 87 low TILs and 58 high TILs patients. There were no significant variations in age and tumor size observed between the low TILs group and high TILs group (P>0.05, **Table 1**). Compared with the low TILs, the tumors with high TILs showed a higher probability of having an oval or round shape, parallel orientation, well-defined margins, complex cystic and solid echo patterns, and posterior enhancement (all p<0.05). The calcification and vascularity exhibited no significant differences between the low TILs group and high TILs group (all p>0.05).

### Univariate analysis of clinical characteristics

In our research, the individuals were randomly allocated into two separate datasets for training and validation purposes, with a distribution ratio of 7:3. No significant differences were found in clinical characteristics between the training and validation datasets (P>0.05), except for margin (P=0.045, **Supplementary Table S1**). There was no difference in age and tumor size between low TILs and high TILs, as observed in the training dataset (P>0.05, **Supplementary Table S2**) and validation dataset (P > 0.05, **Supplementary Table S3**). The training dataset showed that shape, posterior echo, margin, and Rad-score differed significantly (P < 0.05, **Supplementary Table S2**), while the validation dataset showed that echo pattern and posterior echo differed significantly between the low TILs group and high TILs group (P < 0.05, **Supplementary Table S3**).

### Rad-score model establishment

For every individual, a total of 837 radiomics features were obtained. After undergoing a process of feature selection, a LASSO regression analysis (**Supplementary Figure S1**) identified and retained 25 radiomics features (**Supplementary Table S4**), which were used to construct the Rad-score in the training dataset. The high TILs group had higher Rad-score than the low TILs group in all participants (P<0.001, **Table 1**), training dataset (P<0.001, **Supplementary Table S2**), and validation dataset (P=0.002, **Supplementary Table S3**). No statistically difference was detected in the Rad-score between the training dataset and validation dataset (P=0.88, **Supplementary Table S1**).

### Clinical model and Clin+RS integrated model establishment

In the clinical model, multivariable logistic regression analysis revealed that shape (oval/round shape, P=0.024) and posterior echo (posterior enhancement, P=0.007) were independent predictors for predicting high TILs in the training dataset (**Table 2**). In the Clin+RS integrated model, multivariable analysis showed that posterior echo enhancement (P=0.041) and Rad-score (P<0.001) were independent predictors for predicting high TILs in the training dataset (**Table 2**).

**Table 2:** Multivariate logistic analysis.

Characteristics	Clinical model			Clin+RS model		
	OR	95%CI	P value	OR	95%CI	P value
Shape					NA	
Oval/round	2.74	1.15- 6.71	0.024		NA	
Irregular		Reference			NA	
Posterior echo						
Enhancement	4.34	1.53-13.64	0.007	3.39	1.08-11.68	0.041
No/Shadowing		Reference			Reference	
Rad-score	NA	NA	NA	7.83	3.21-24.17	<0.001

Clin+RS, clinical features plus Rad-score.

NA, None applicable.

### Performance, construction, and validation of nomogram

**Figure 3:**
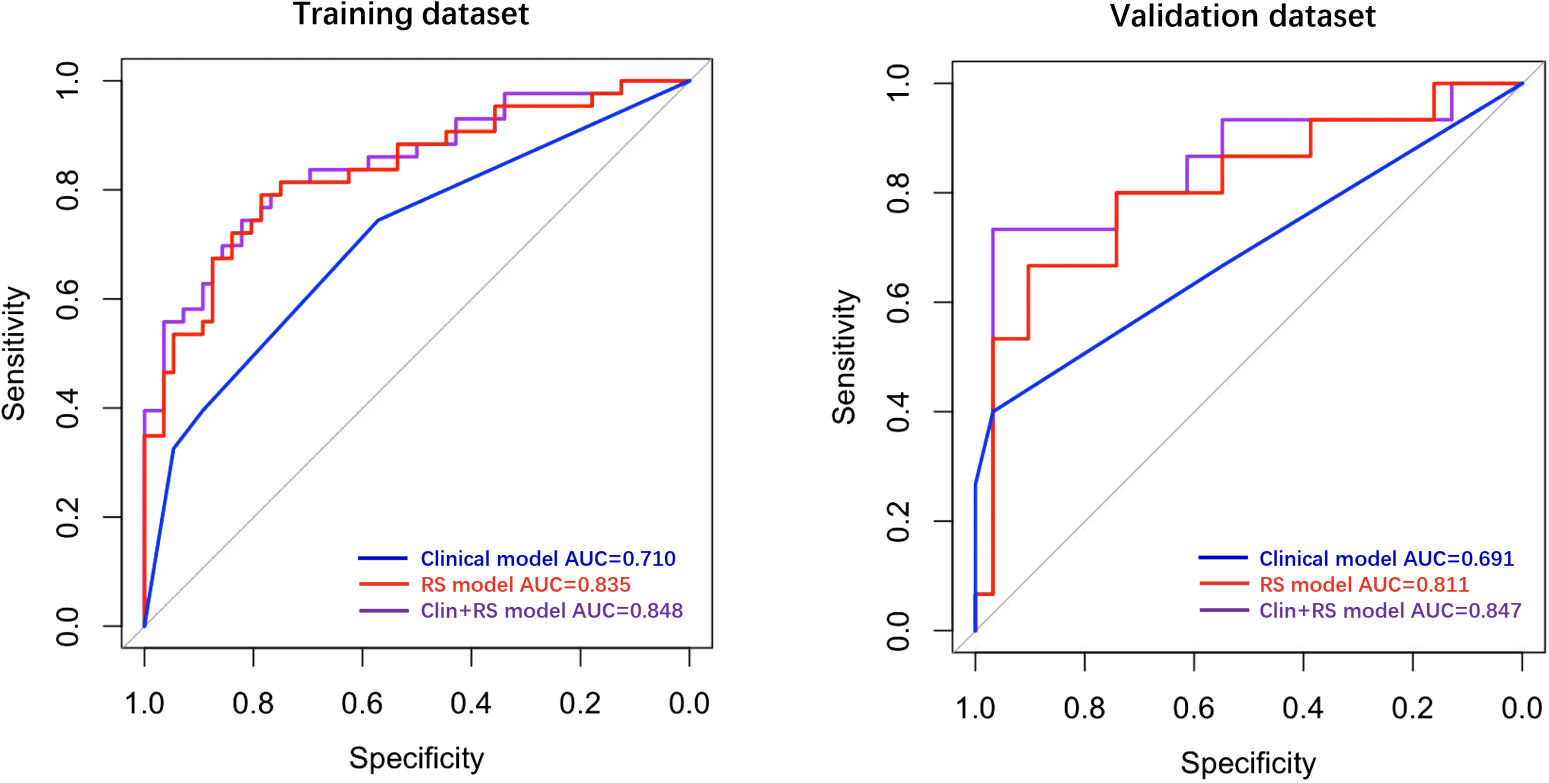
ROC curves for predicting high TILs in training and validation datasets. Clin+RS, clinical+Rad-score; ROC, Receiver operating characteristic; RS, Rad-score.

**Figure 4:**
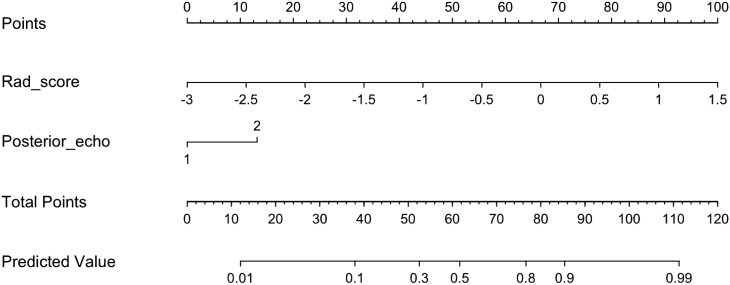
Nomogram for the clinical+Rad-score model for predicting the probability of high TILs.

**Figure 5:**
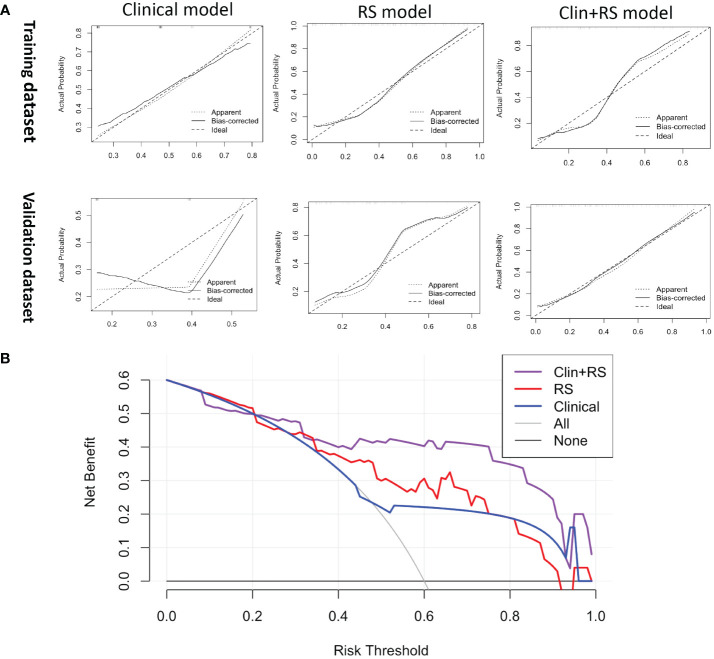
**(A)** The calibration curve of the clinical, RS, and Clin+RS models for predicting the probability of high TILs. **(B)** DCA for predicting the probability of high TILs in the validation dataset. Clin+RS, clinical features plus Rad-score; DCA, Decision curve analysis; RS, Rad-score.

In the training dataset, the clinical model achieved an AUC of 0.710, while the Rad-score model showed a higher AUC of 0.835. The Clin+RS integrated model outperformed both with an even higher AUC of 0.848 (**Figure 3**, **Table 3**). Compared to the clinical model, the Rad-score model (P=0.039), and Clin+RS integrated model (P=0.007) showed better performances; compared to the Rad-score model, Clin+RS integrated model showed better performance (P=0.048). In the validation dataset, the clinical model achieved an AUC of 0.691, while the Rad-score model showed an improved performance with an AUC of 0.811. Notably, when integrating both clinical and Rad-score features in the Clin+RS model, a higher AUC of 0.847 was observed (**Figure 3**, **Table 3**). A nomogram of the Clin+RS integrated model was further developed to individually predict high TILs in the patients with TNBC (**Figure 4**). In the validation dataset, the prediction results and observations showed a strong correspondence in terms of calibration and DCA curves for the Clin+RS integrated model (**Figure 5**).

**Table 3:** Diagnostic performance of the prediction models in the training and validation datasets.

Prediction models	Datasets	AUC (95%CI)	Accuracy	Sensitivity	Specificity
Clinical model	Training	0.710	0.646	0.744	0.571
Clinical model	Validation	0.691	0.783	0.40	0.968
Rad-score model	Training	0.835	0.788	0.791	0.786
Rad-score model	Validation	0.811	0.826	0.667	0.903
Clin+RS model	Training	0.848	0.788	0.744	0.821
Clin+RS model	Validation	0.847	0.891	0.733	0.968

Clin+RS, clinical features plus Rad-score.

## Discussion

In the current research, we effectively created and verified a nomogram that combines CUS characteristics with CUS-Radiomics features. The nomogram functions as an evaluation tool for preoperatively assessing TIL levels in patients with TNBC, offering valuable perspectives without requiring invasive procedures. Moreover, the nomogram derived from the Clin+RS integrated model demonstrated enhanced predictive accuracy and net benefit compared to the clinical or Rad-score models individually.

Current treatment strategies for individuals with breast cancer predominantly rely on the molecular categorization of this disease (27, 28). TNBC, which accounts for approximately 10% to 20% of cases with breast cancer, poses difficulties in treatment due to its heterogeneous nature and absence of clearly defined molecular targets (29, 30). Moreover, TNBC is linked to an increased likelihood of recurrence and metastasis, leading to unfavorable consequences (29). TILs have been identified as indicators of immune infiltration and prognosis in breast cancer, showing promise as potential biomarkers for predicting patient response to immunotherapy (3, 4). However, the current quantification of TILs relies on the manual assessment using pathological slides, which is restricted by the invasive nature of specimen collection and the labor-intensive analysis method. Therefore, noninvasive preoperative imaging examinations are crucial for predicting the TIL level in TNBC as a supplementary diagnostic method.

CUS has been proved to be a dependable imaging technique in the clinical diagnosis of breast cancer. The present research established a clinical model to determine the TIL level using independent predictors of tumor shape and posterior echo features. In the clinical model, we found that the tumors with high TILs had a higher probability of have an oval/round shape and posterior echo enhancement, which are the characteristics of benign breast lesions (31, 32). Indeed, many TNBC tumors are easily misdiagnosed as benign tumors because of their benign-like CUS appearances (33). Therefore, the tumors with high TILs may be more likely to be misdiagnosed as benign tumors, such as fibroadenomas. The biological mechanism of the association between these sonographic appearances and the TIL level remains to be elucidated.

Radiomics has become increasingly significant in the diagnosis and prognosis prediction, and the Rad-score model has been proposed for assessing the therapeutic outcome and prognosis of individuals diagnosed with breast cancer (21, 22). In this study, after radiomics feature selection, 25 radiomics features that described the tumor heterogeneity were associated with the TIL level (**Supplementary Table S4**). Then, the Rad-score was created using a set of 25 radiomics features mentioned above. Furthermore, we established three models (clinical model, Rad-score model and Clin+RS integrated model) to noninvasively predict the TIL level. Of these three models, the Clin+RS integrated model demonstrated superior performance, achieving an AUC of 0.848 and 0.847 in the training dataset and validation dataset correspondingly. Prior to this study, multiple investigations have investigated the correlation between radiomics and TILs using mammographic or MRI images in patients with breast cancer (34–38). However, CUS is the most commonly diagnostic imaging method for breast tumors in clinical settings due to the real-time, non-radiative, and cost-effective advantages. To the best of our knowledge, our research is the first to investigate the correlation between radiomics and TILs in TNBC utilizing CUS images. The validation cohort demonstrated excellent performance of the prediction model, achieving an AUC value of 0.847. Furthermore, the prediction model was validated by its high accuracy (0.891), sensitivity (0.733), and specificity (0.968). The calibration curve of the integrated model demonstrated a favorable concordance, while the DCA of the integrated model indicated a greater net benefit. Thus, the Clin+RS integrated model has the potential to serve as a reliable and valuable approach in effectively differentiating between high and low TILs in patients with TNBC. In addition, nomogram is a useful tool for multi-index joint diagnosis or prediction in breast cancers, providing substantial advantages in clinical settings offering (39, 40). The present study further constructed a Clin+RS integrated nomogram for preoperatively predicting the TIL level in TNBC.

This study has some limitations. First, our study conducted a retrospective analysis at a single institution with a restricted sample size. In the future, the model needs to be further validated by larger sample sizes from multiple centers and a prospective cohort. Second, we only analyzed CUS-based radiomics features. Future studies could further perform radiomics analyses using ultrasound elastography and contrast-enhanced ultrasound images. Lastly, the variations in ultrasound image acquisition stemming from different ultrasound equipment may also influence experimental outcomes.

In summary, the radiomics features based on CUS exhibit promising potential as a biomarker for differentiation between high and low TILs in patients with TNBC. The Clin+RS integrated model, which integrates posterior echo and Rad-score, exhibits enhanced predictive precision in comparison to the clinical or Rad-score models individually. In addition, the Clin+RS integrated nomogram holds tremendous potential for preoperative individualized prediction of the TIL level in TNBC.

## Data availability statement

The original contributions presented in the study are included in the article/**Supplementary Material**. Further inquiries can be directed to the corresponding authors.

## Ethics statement

The studies involving humans were approved by the ethics consultant committee of Second Affiliated Hospital, Zhejiang University School of Medicine. The studies were conducted in accordance with the local legislation and institutional requirements. The participants provided their written informed consent to participate in this study.

## Author contributions

LH: Conceptualization, Data curation, Formal analysis, Funding acquisition, Investigation, Methodology, Software, Writing – original draft. PJ: Data curation, Formal analysis, Funding acquisition, Investigation, Methodology, Resources, Software, Validation, Writing – original draft. WX: Data curation, Formal analysis, Investigation, Resources, Writing – original draft. CW: Conceptualization, Project administration, Supervision, Visualization, Writing – review & editing. PH: Funding acquisition, Project administration, Resources, Supervision, Writing – review & editing.
