# Supplementary material for: Clinical and radiomics integrated nomogram for preoperative prediction of tumor-infiltrating lymphocytes in patients with triple-negative breast cancer
Source: Front Oncol. 2024 Mar 19;14:1370466. doi: 10.3389/fonc.2024.1370466 (PMC10985173; doi:10.3389/fonc.2024.1370466)
Supplement: Supplementary file 1 [file DataSheet_1.docx]

**Rad-score calculation formula**

Rad-score= (-7.15E+02)+(-1.86E-01) *exponential_glcm_Correlation

| +(6.47E-02)* exponential_glcm_Imc1 |
| --- |
| +(4.54E-01)* exponential_glrlm_RunLengthNonUniformityNormalized |
| +(4.37E-01) * exponential_glrlm_ShortRunLowGrayLevelEmphasis |
| +(4.73E-15) * exponential_glszm_SmallAreaLowGrayLevelEmphasis |
| +(-2.93E-01) * exponential_ngtdm_Strength |
| +(-6.61E-02) * gradient_firstorder_Maximum |
| + (-3.35E-16) * gradient_firstorder_Range |
| + (7.02E-01) * gradient_glcm_Correlation |
| +(1.61E-01) * gradient_ngtdm_Busyness |
| +(-7.94E-01) * lbp.2D_firstorder_InterquartileRange |
| +(-7.12E-01) * lbp.2D_firstorder_Variance |
| +(1.43E-01) * lbp.2D_glrlm_RunLengthNonUniformity |
| +(2.46E-01) * lbp.2D_glrlm_ShortRunEmphasis |
| +(8.07E-16) * lbp.2D_glrlm_ShortRunLowGrayLevelEmphasis |
| +(1.86E-02) * lbp.2D_glszm_SizeZoneNonUniformity |
| +(8.94E-04) * lbp.2D_glszm_SmallAreaEmphasis |
| +(2.72E-04) * lbp.2D_glszm_SmallAreaHighGrayLevelEmphasis |
| +(6.73E-17) * lbp.2D_glszm_SmallAreaLowGrayLevelEmphasis |
| + (-1.64E+00) * logarithm_firstorder_Maximum |
| +(6.95E+02) * square_glcm_Idmn |
| +(5.28E+01) * square_glcm_Idn |
| +(3.16E-02) * square_glcm_MaximumProbability |
| +(-2.96E+01) * wavelet.H_firstorder_Entropy |
| +(3.20E-01) * wavelet.H_firstorder_Skewness |

**Table S1.** **Comparisons of clinical characteristics and Rad-score between training and validation datasets.**

| **Characteristics** | **Training dataset**  **(n=99)** | **Validation dataset**  **(n=46)** | **P value** |
| --- | --- | --- | --- |
| Age (y), mean ± SD | 53.9±9.9 | 56.3±11.2 | 0.214 |
| Size (mm), median | 20.0 (15, 25) | 19.5 (15, 25) | 1 |
| Shape |  |  | 0.715 |
| Oval/round | 50 (51%) | 21 (46%) |  |
| Irregular | 49 (49%) | 25 (54%) |  |
| Orientation |  |  | 0.469 |
| Parallel | 80 (81%) | 34 (74%) |  |
| Not parallel | 19 (19%) | 12 (26%) |  |
| Margin |  |  | 0.045 |
| Well-defined | 35 (35%) | 8 (17%) |  |
| Ill-defined | 64 (65%) | 38 (83%) |  |
| Echo pattern |  |  | 0.465 |
| Complex cystic-solid | 94 (95%) | 42 (91%) |  |
| Hypoechoic | 5 (5%) | 4 (9%) |  |
| Posterior echo |  |  | 0.374 |
| Enhancement | 23 (23%) | 7 (15%) |  |
| No/Shadowing | 76 (77%) | 39 (85%) |  |
| Calcification |  |  | 1 |
| Absent | 37 (37%) | 17 (37%) |  |
| Present | 62 (63%) | 29 (63%) |  |
| Vascularity |  |  | 0.672 |
| Absent | 24 (24%) | 14 (30%) |  |
| Internal vascularity | 46 (46%) | 21 (46%) |  |
| Vessels in rim | 29 (29%) | 11 (24%) |  |
| Rad-score, median | -0.53 (-1.01, -0.14) | -0.45 (-1.17, -0.16) | 0.88 |

**Table S2.** **Univariate analysis of clinical characteristics and Rad-score in the training dataset.**

| **Characteristics** | **Low TILs**  **(n=56)** | **High TILs**  **(n=43)** | **P value** |
| --- | --- | --- | --- |
| Age (y), mean ± SD | 54.5 (50.7, 60.2) | 53 (47.5, 56.5) | 0.035 |
| Size (mm), median | 20 (15, 25) | 21 (15.5, 25) | 0.252 |
| Shape |  |  | 0.006 |
| Oval/round | 21 (38%) | 29 (67%) |  |
| Irregular | 35 (62%) | 14 (33%) |  |
| Orientation |  |  | 0.367 |
| Parallel | 43 (77%) | 37 (86%) |  |
| Not parallel | 13 (23%) | 6 (14%) |  |
| Margin |  |  | 0.008 |
| Well-defined | 13 (23%) | 22 (51%) |  |
| Ill-defined | 43 (77%) | 21 (49%) |  |
| Echo pattern |  |  | 0.65 |
| Complex cystic-solid | 54 (96%) | 40 (93%) |  |
| Hypoechoic | 2 (4%) | 3 (7%) |  |
| Posterior echo |  |  | 0.002 |
| Enhancement | 6 (11%) | 17 (40%) |  |
| No/Shadowing | 50 (89%) | 26 (60%) |  |
| Calcification |  |  | 0.549 |
| Absent | 19 (34%) | 18 (42%) |  |
| Present | 37 (66%) | 25 (58%) |  |
| Vascularity |  |  | 0.688 |
| Absent | 14 (25%) | 10 (23%) |  |
| Internal vascularity | 24 (43%) | 22 (51%) |  |
| Vessels in rim | 18 (32%) | 11 (26%) |  |
| Rad-score, median | -0.79 (-1.25, -0.52) | -0.14 (-0.45, 0.4) | < 0.001 |

**Table S3.** **Univariate analysis of clinical characteristics and Rad-score in the validation dataset.**

| **Characteristics** | **Low TILs**  **(n=31)** | **High TILs**  **(n=15)** | **P value** |
| --- | --- | --- | --- |
| Age (y), mean ± SD | 55.4±11.8 | 58.3±10.0 | 0.389 |
| Size (mm), median | 19 (13, 25) | 20 (16.5, 27.5) | 0.318 |
| Shape |  |  | 0.68 |
| Oval/round | 13 (42%) | 8 (53%) |  |
| Irregular | 18 (58%) | 7 (47%) |  |
| Orientation |  |  | 0.07 |
| Parallel | 20 (65%) | 14 (93%) |  |
| Not parallel | 11 (35%) | 1 (7%) |  |
| Margin |  |  | 0.092 |
| Well-defined | 3 (10%) | 5 (33%) |  |
| Ill-defined | 28 (90%) | 10 (67%) |  |
| Echo pattern |  |  | 0.008 |
| Complex cystic-solid | 31 (100%) | 11 (73%) |  |
| Hypoechoic | 0 (0%) | 4 (27%) |  |
| Posterior echo |  |  | 0.003 |
| Enhancement | 30 (97%) | 9 (60%) |  |
| No/Shadowing | 1 (3%) | 6 (40%) |  |
| Calcification |  |  | 1 |
| Absent | 11 (35%) | 6 (40%) |  |
| Present | 20 (65%) | 9 (60%) |  |
| Vascularity |  |  | 0.207 |
| Absent | 12 (39%) | 2 (13%) |  |
| Internal vascularity | 13 (42%) | 8 (53%) |  |
| Vessels in rim | 6 (19%) | 5 (33%) |  |
| Rad-score, median | -0.86±0.71 | 0.09±0.93 | 0.002 |


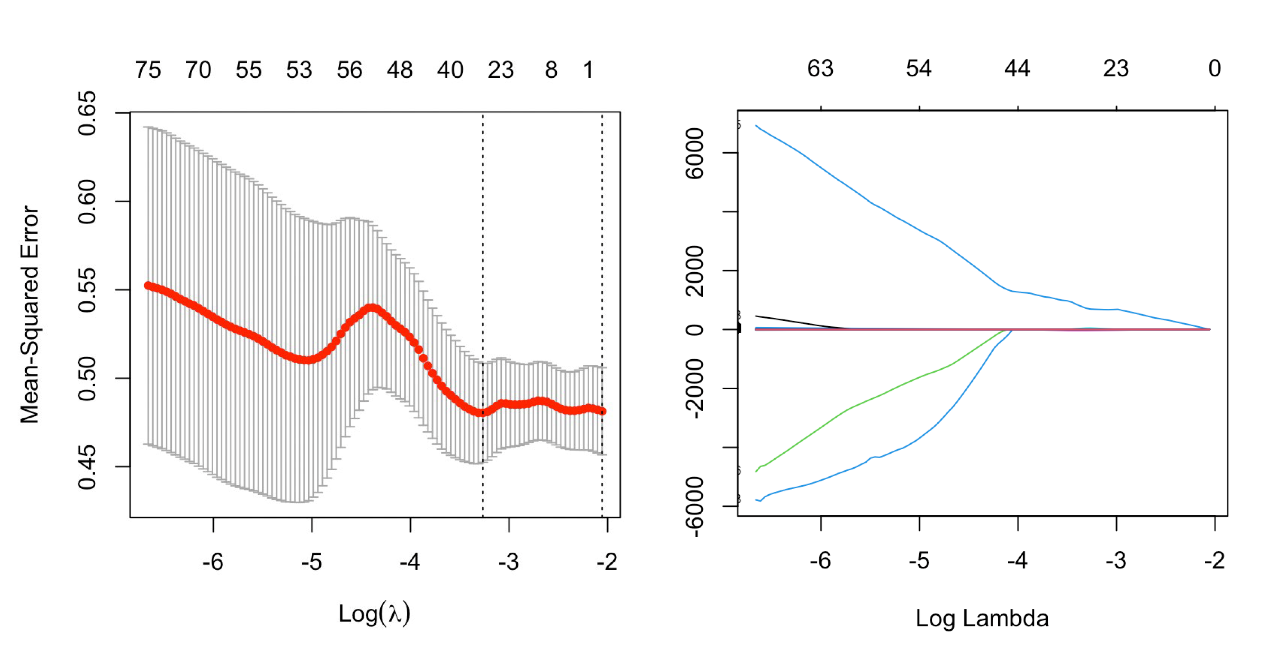


**Fugire S1.** The results of LASSO for radiomics features selection.

**Table S4. Selected radiomics features**

| **Radiomics features** **classes** | **Radiomics features** |
| --- | --- |
| First-order (n=7) | gradient_firstorder_Maximum  gradient_firstorder_Range  lbp.2D_firstorder_InterquartileRange  lbp.2D_firstorder_Variance  logarithm_firstorder_Maximum  wavelet.H_firstorder_Entropy  wavelet.H_firstorder_Skewness |
| GLCM (n=6) | exponential_glcm_Correlation  exponential_glcm_Imc1  gradient_glcm_Correlation  square_glcm_Idmn  square_glcm_Idn  square_glcm_MaximumProbability |
| GLRLM (n=5) | exponential_glrlm_RunLengthNonUniformityNormalized  exponential_glrlm_ShortRunLowGrayLevelEmphasis  lbp.2D_glrlm_RunLengthNonUniformity  lbp.2D_glrlm_ShortRunEmphasis  lbp.2D_glrlm_ShortRunLowGrayLevelEmphasis |
| GLSZM (n=5) | exponential_glszm_SmallAreaLowGrayLevelEmphasis  lbp.2D_glszm_SizeZoneNonUniformity  lbp.2D_glszm_SmallAreaEmphasis  lbp.2D_glszm_SmallAreaHighGrayLevelEmphasis  lbp.2D_glszm_SmallAreaLowGrayLevelEmphasis |
| NGTDM (n=2) | exponential_ngtdm_Strength  gradient_ngtdm_Busyness |

GLCM, gray-level co-occurrence matrix, GLSZM, gray-level size zone matrix, GLRLM, gray-level run length matrix, NGTDM, neighboring gray tone difference matrix.
